# Higher Energy and Zinc Intakes from Complementary Feeding Are Associated with Decreased Risk of Undernutrition in Children from South America, Africa, and Asia

**DOI:** 10.1093/jn/nxaa271

**Published:** 2020-09-16

**Authors:** Bruna L L Maciel, Priscila N Costa, José Q Filho, Samilly A Ribeiro, Francisco A P Rodrigues, Alberto M Soares, Francisco S Júnior, Ramya Ambikapathi, Elizabeth T R McQuade, Margaret Kosek, Tahmeed Ahmed, Pascal Bessong, Gangadeep Kang, Sanjaya Shresthra, Estomih Mduma, Eliwaza Bayo, Richard L Guerrant, Laura E Caulfield, Aldo A M Lima

**Affiliations:** Nutrition Postgraduation Program, Department of Nutrition, Federal University of Rio Grande do Norte, Natal, Brazil; Nutrition Postgraduation Program, Department of Nutrition, Federal University of Rio Grande do Norte, Natal, Brazil; INCT—Instituto de Biomedicina do Semiárido Brasileiro(IBISAB), Faculty of Medicine, Federal University of Ceará, Fortaleza, Brazil; INCT—Instituto de Biomedicina do Semiárido Brasileiro(IBISAB), Faculty of Medicine, Federal University of Ceará, Fortaleza, Brazil; INCT—Instituto de Biomedicina do Semiárido Brasileiro(IBISAB), Faculty of Medicine, Federal University of Ceará, Fortaleza, Brazil; INCT—Instituto de Biomedicina do Semiárido Brasileiro(IBISAB), Faculty of Medicine, Federal University of Ceará, Fortaleza, Brazil; INCT—Instituto de Biomedicina do Semiárido Brasileiro(IBISAB), Faculty of Medicine, Federal University of Ceará, Fortaleza, Brazil; Department of Public Health, Purdue University, West Lafayette, IN, USA; Department of Public Health Sciences, University of Virginia, Charlottesville, VA, USA; Department of International Health, The Johns Hopkins Bloomberg School of Public Health, Baltimore, MD, USA; Division of Nutrition and Clinical Services, icddr,b, Dhaka, Bangladesh; University of Venda, Thohoyandou, South Africa; Division of Gastrointestinal Sciences, Christian Medical College, Vellore, India; Walter Reed/Armed Forces Research Institute of Medical Sciences (AFRIMS) Research Unit, Nepal (WARUN), Kathmandu, Nepal; Haydom Global Health Research Centre, Haydom Lutheran Hospital, Haydom, Tanzania; Haydom Global Health Research Centre, Haydom Lutheran Hospital, Haydom, Tanzania; Center for Global Health, Division of Infectious Diseases and International Health, University of Virginia School of Medicine, Charlottesville, VA, USA; Center for Human Nutrition, Department of International Health, The Johns Hopkins Bloomberg School of Public Health, Baltimore, MD, USA; INCT—Instituto de Biomedicina do Semiárido Brasileiro(IBISAB), Faculty of Medicine, Federal University of Ceará, Fortaleza, Brazil

**Keywords:** child nutrition, dietary intake, nutrient intake, energy, zinc

## Abstract

**Background:**

Few studies have focused on quantitatively analyzing nutrients from infant diets, compromising complementary feeding evaluation and health promotion worldwide.

**Objectives:**

This study aimed to describe dietary intake in infants from 9 to 24 mo of age, determining nutrient intakes associated with the risk of underweight, wasting, and stunting.

**Methods:**

Usual nutrient intakes from complementary feeding were determined by 24-h recalls collected when infants were 9–24 mo of age in communities from 7 low- and middle-income countries: Brazil (*n* = 169), Peru (*n* = 199), South Africa (*n* = 221), Tanzania (*n* = 210), Bangladesh (*n* = 208), India (*n* = 227), and Nepal (*n* = 229), totaling 1463 children and 22,282 food recalls. Intakes were corrected for within- and between-person variance and energy intake. Multivariable regression models were constructed to determine nutrient intakes associated with the development of underweight, wasting, and stunting at 12, 18, and 24 mo of age.

**Results:**

Children with malnutrition presented significantly lower intakes of energy and zinc at 12, 18, and 24 mo of age, ranging from −16.4% to −25.9% for energy and −2.3% to −48.8% for zinc. Higher energy intakes decreased the risk of underweight at 12 [adjusted odds ratio (AOR): 0.90; 95% CI: 0.84, 0.96] and 24 mo (AOR: 0.91; 95% CI: 0.86, 0.96), and wasting at 18 (AOR: 0.91; 95% CI: 0.83, 0.99) and 24 mo (AOR: 0.83; 95% CI: 0.74, 0.92). Higher zinc intakes decreased the risk of underweight (AOR: 0.12; 95% CI: 0.03, 0.55) and wasting (AOR: 0.19; 95% CI: 0.04, 0.92) at 12 mo, and wasting (AOR: 0.05; 95% CI: 0.00, 0.76) at 24 mo.

**Conclusions:**

Higher intakes of energy and zinc in complementary feeding were associated with decreased risk of undernutrition in the studied children. Data suggest these are characteristics to be improved in children's complementary feeding across countries.

See corresponding commentary on page 5.

## Introduction

Infant feeding practices directly affect the nutritional status of children and child survival (1). The time between birth and 2 y of age is critical for health, development, and stunting prevention (2, 3). Breastfeeding and complementary feeding practices determine nutritional status, growth, and development, and imprint physiologic and metabolic mechanisms that lower the risk of infectious diseases (4).

The risk of undernutrition during the first 2 y of life increases during the complementary feeding period (5, 6). Insufficient quantities and poor quality of complementary foods, together with inadequate feeding practices and increased rates of infection during this period, are potential risk factors for stunting (7, 8).

Although breastfeeding practices have been evaluated continuously and promoted through research and policies, complementary feeding evaluation and thus promotion have encountered several limitations. The WHO core indicators (1), standardized measures for complementary feeding quality assessment, represent simple indicators to make complementary feeding assessment more feasible worldwide. Studies have addressed characterizing complementary feeding in local communities (9), some using part of the WHO indicators (10) and their relation with nutritional status (9, 11, 12), but none were able to determine usual nutrient intake from complementary feeding as a determinant of nutritional status. Quantitative estimation of food and nutrient intake presents field difficulties, considering that food recalls are complicated, requiring prospective study designs for reliability. These limitations have restricted cross-country evaluation of complementary feeding characteristics, especially concerning nutrient intake estimates. Understanding which nutrient intakes from complementary feeding are associated with malnutrition is important especially across countries, considering different cultures’ impact on dietary access and diversity.

The MAL-ED (Etiology, Risk Factors and Interactions of Enteric Infections and Malnutrition and the Consequences for Child Health and Development) study is a longitudinal birth cohort study in 8 low- or middle-income countries (13). In this study, food recalls were prospectively collected from 9 to 24 mo of age (14). These data enable the characterization of different nutrient intakes associated with the risk of underweight, wasting, and stunting in infants. This study aimed to describe dietary intake of infants from 9 to 24 mo of age, determining nutrient intakes associated with the risk of underweight, wasting, and stunting. We hypothesized that lower energy and nutrient intakes from complementary feeding of children from 9 to 24 mo of age would increase the risk of undernutrition during this period.

## Methods

### Ethics approval

The study was approved by the Institutional Review Board for Health Sciences Research, University of Virginia, USA as well as the respective governmental, local institutional, and collaborating institutional ethical review boards at each site: Committee for Ethics in Research, Universidade Federal do Ceará; National Ethical Research Committee, Health Ministry, Council of National Health in Brasília and Fortaleza, Brazil (Brazilian site); Institutional Review Board, Johns Hopkins University, in Baltimore, MD, USA; PRISMA Ethics Committee; Health Ministry, in Loreto, Peru (Peruvian site); Health, Safety and Research Ethics Committee, University of Venda; Department of Health and Social Development, Limpopo Provincial Government, in Venda, South Africa (South African site); Medical Research Coordinating Committee, National Institute for Medical Research; Chief Medical Officer, Ministry of Health and Social Welfare in Haydom, Tanzania (Tanzanian site); Ethical Review Committee, icddr,b in Dhaka, Bangladesh (Bangladesh site); Institutional Review Board, Christian Medical College in Vellore, India and the Health Ministry Screening Committee, Indian Council of Medical Research (Indian site); and Institutional Review Board, Institute of Medicine, Tribhuvan University; Ethical Review Board, Nepal Health Research Council; Institutional Review Board, Walter Reed Army Institute of Research in Bhaktapur, Nepal (Nepalese site). Informed written consent was obtained from the parent or legal guardian of each participating child.

### Study population

**FIGURE 1:**
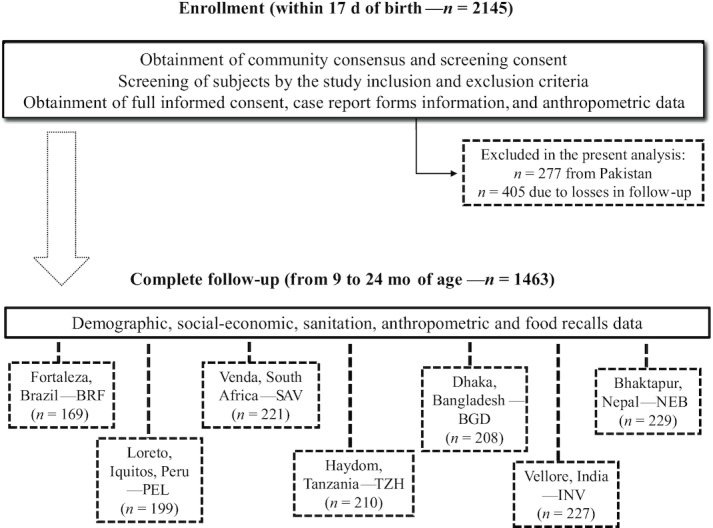
Flow diagram of the study protocol.

In the present analysis, the MAL-ED data from enrollment to 24 mo were used. Data were collected from 2010 to 2014. By 24 mo of age, complete data from 1463 children in poor communities from 7 low- and middle-income countries were available: Fortaleza, Brazil (BRF) (*n* = 169); Loreto, Iquitos, Peru (PEL) (*n* = 199); Venda, South Africa (SAV) (*n* = 221); Haydom, Tanzania (TZH) (*n* = 210); Dhaka, Bangladesh (BGD) (*n* = 208); Vellore, India (INV) (*n* = 227); and Bhaktapur, Nepal (NEB) (*n* = 229). These communities were in urban (Brazil, Bangladesh, India, Nepal), peri-urban (South Africa), and rural contexts (Peru and Tanzania). Although Naushahro Feroze, Pakistan (PKN) was part of the MAL-ED, data from this site are not used here owing to measurement quality concerns (**Figure 1**).

Each site made a census of their community to obtain an assessment of the number of women of reproductive age and the number of children <5 y of age. From these data, a catchment area was defined in each site where it was estimated that >200 infants would be born within the enrollment period lasting 24 mo. Inclusion criteria in the study were healthy singleton newborn enrolled within 17 d of birth; birth weight >1500 g; child from a family intending to stay in the study area for the next 6 mo; no other child from the same family enrolled in the study; and mother aged 16 y or older. Children were excluded from the study if they had congenital diseases, severe diseases that required hospitalization, or any other condition that was severe or chronic such as renal disease, chronic heart failure, or severe liver disease (13).

### Anthropometric and dietary data collection

Anthropometric measurements were collected on all children at enrollment and then monthly using standardized procedures (13). Training of experienced field workers for anthropometric measures was performed for each site. Sites used instruments that met technical specifications, as recommended by the WHO (15). Every week, the scale in each site was checked/calibrated with suitable standards. Each month, a supervisor or highly trained study staff member collected a set of duplicate anthropometric measurements for 5% of the participants within 24 h of the monthly data collection. Reliability estimates (*r*) for both weights and lengths were >0.9, and all quality control procedures are described by Richard et al. (16). Underweight, wasting, and stunting were defined according to the WHO recommendations (17), using the weight-for-age, weight-for-length, and length-for-age *z* scores.

Nutritional surveillance was conducted through home visits twice weekly, during which the caregiver reported (yes or no) the child's consumption in the previous 24 h of breast milk, animal milk, formula, other liquids, water, tea, fruit juice, semisolids, and specific solid foods (14). Breastfeeding status at each visit was characterized as exclusive, predominant, partial, or none (18).

Beginning at 9 mo and monthly thereafter, the caregiver was asked to recall non-breast-milk food intake over the prior 24 h to estimate the energy and nutrient intake of the child using the 24-h recall method. These dietary data were collected monthly (starting at 9 mo) by trained fieldworkers, using forms completed by hand, and included details on the ingredients and preparation steps (i.e., recipe) for complex local preparations.

The sites used the same 24-h recall form for data collection. Training for using the 24-h recall technique was conducted by the Nutrition Technical Subcommittee for each study site following general principles (19). Each site developed appropriate tools to aid in quantifying amounts and recipes, as previously described by Caulfield et al. (14). To enhance the data collected each month, ∼10–15 children were randomly selected to have a secondary recall, 2–7 d after the monthly recall. A randomization procedure was performed, and each child was randomly allocated to have a secondary recall done after 1 study visit between 9 and 24 mo of age. This procedure made 15–17 recalls per child available, and we analyzed 22,282 food recalls in the present study.

Data were double-entered into a computer and transmitted to the Data Coordinating Center, Bethesda, MD. Multiple searches to identify errors and retraining of field staff based on error identification were performed with researchers from the Johns Hopkins University Bloomberg School of Public Health (JHSPH). Communication with the researchers at JHSPH resolved issues or questions identified during data collection. Then, energy and nutrient analyses were performed at JHSPH, using site-specific food composition tables created for the study in Excel 2013 (Microsoft) (14, 20, 21). For the present analysis, intakes of energy, macronutrients, fiber, 6 vitamins (niacin, riboflavin, thiamin, folate, and vitamins A and C), and 6 minerals (calcium, iron, magnesium, potassium, phosphorus, and zinc) were used. Usual dietary intake from complementary feeding was determined considering the 24-h recalls from 9 to 24 mo of age for each child, as detailed in data analysis.

### Enteropathogen detection

Environmental enteropathy, caused by chronic enteric infections, is a leading cause of high rates of stunting and growth failure (22, 23). Thus, enteropathogen burdens were used in the present analysis as adjustment variables in the models. Nondiarrheal stool samples were collected monthly and tested for 29 enteropathogens using qPCR with custom-designed TaqMan Array Cards (ThermoFisher), as previously described (24, 25). These pathogens were bacteria [*Campylobacter* spp., *Shigella*, enteroaggregative *Escherichia coli*(EAEC), typical enteropathogenic *Escherichia coli*(tEPEC), atypical enteropathogenic *Escherichia coli* (aEPEC), enterotoxigenic *Escherichia coli* (ETEC), Shiga toxin-producing *Escherichia coli* (STEC), *Aeromonas, Helicobacter pylori, Plesiomonas, Salmonella*, and *Vibrio cholerae*), viruses (rotavirus, norovirus, adenovirus 40/41, astrovirus, and sapovirus), and parasites (*Cryptosporidium, Giardia, Enterocytozoon bieneusi, Trichuris, Encephalitozoon intestinalis, Cyclospora, Isospora, Entamoeba histolytica, Acyclostoma, Ascaris, Necator*, and *Strongyloides*]. A sample was positive for a pathogen when the qPCR cycle threshold was <35, the analytic limit of detection. The total number of bacteria, viruses, and parasites detected in each stool sample was calculated, and the mean number of pathogens in each group detected between 9 and 24 mo of age was used to characterize subclinical pathogen burden during this age period.

### Data analysis

Data were double entered by trained personnel. Consistency checks and data cleaning were accomplished. Categorical variables were tested using the chi-square test. Quantitative variables were tested for normality using the Kolmogorov–Smirnov test. The Kruskal–Wallis test was used to compare nonparametric variables across the studied sites. Usual intake of nutrients was estimated in 3 periods using the 24-h recalls from 9–12 mo, 13–18 mo, and 19–24 mo. Nutrient intakes with asymmetric distributions were approximated to normal distributions using the square root. One-factor ANOVA was used to estimate the within- and between-person variance based on the quadratic means from the ANOVA output. Then, back transformation of the corrected values (in square root) was performed to return the estimated usual intakes to their original scales. In order to control for confounding factors inherent in total energy intake, adjustment of the nutrient intake by energy using the residual method was done (26). Briefly, energy adjustment was performed by regression in which each absolute nutrient intake (corrected by ANOVA) was the dependent variable and the total energy intake (corrected by ANOVA) the independent variable; the residuals from each model were then translated back to their original units.

Nutrient intakes are presented as median intakes and IQRs in the 3 time periods. The Mann–Whitney *U* test was used to compare energy and nutrient intakes between the children with or without underweight, wasting, and stunting. The differences in intake between the children with or without underweight, wasting, and stunting were expressed as percentages considering children without underweight, wasting, and stunting as the reference value.

Logistic regressions were constructed first in bivariate analyses, exploring the effect of a single nutrient intake on the outcomes of underweight, wasting, and stunting at 12, 18, and 24 mo, and crude ORs and 95% CIs were calculated. Then, 3 multivariable logistic regression models were constructed for each time period, considering as outcomes the presence of underweight, wasting, or stunting at 12, 18, and 24 mo. Because infections compromise nutritional status (27, 28), bacterial, viral, and parasite burdens were included in the models as independent adjustment variables. Sex, country of the site, length-for-age at enrollment (within 17 d of birth), and breastfeeding at 12, 18, or 24 mo (yes/no) were also included as adjustment variables. Adjusted nutrient intakes were approximated to normal distributions before entering the models using their square root. Multicollinearity between nutrient intakes was assessed, and those showing correlation (Pearson's *r* > 0.7) were excluded from the adjusted models. Final model adjustment was observed through the Omnibus tests of model coefficients, with *P* values < 0.05 considered significant. The Hosmer–Lemeshow test was also used, considering *P* values > 0.05 as reliable. Adjusted odds ratios (AORs) and 95% CIs were shown to assess the risk association found between nutrient intake and the outcome analyzed in the model. The analysis was performed using SPSS version 23 (IBM).

## Results

Underweight, wasting, and stunting were present in *n* = 202 (14.0%), *n* = 76 (5.3%), and *n* = 384 (26.6%) of the studied children at 12 mo, respectively; *n* = 230 (15.9%), *n* = 68 (4.7%), and *n* = 545 (37.9%) of the children at 18 mo, respectively; and *n* = 257 (17.9%), *n* = 63 (4.4%), and *n* = 551 (38.4%) at 24 mo, respectively (**Table 1**). At 24 mo of age, TZH, BGD, INV, and NEB presented higher prevalences of undernutrition. For underweight, INV and BGD presented a higher prevalence, of 36.3% and 32.7%, respectively. NEB presented a higher prevalence of wasting (12.8%), followed by INV (11.5%). Stunting was more prevalent in BGD (48.8%), followed by INV (44.2%) (Table 1). Although gender was not different in the study populations, stunting was significantly more prevalent in boys (57.5%) than in girls at 12 (59.1% compared with 40.9%), 18 (55.3% compared with 44.7%), and 24 (57.5% compared with 42.5%) months of age (chi-square test, *P* < 0.001).

**TABLE 1:** Length-for-age at enrollment, and breastfeeding and the presence of undernutrition from 12 to 24 mo of age, in children from the studied MAL-ED sites^1^

Variables	Total	BRF	PEL	SAV	TZH	BGD	INV	NEB
Length-for-age at enrollment—within 17 d of birth (*z* scores), median [IQR]^2^	−0.36 [−1.1 to 0.58]	−0.54 [−1.2 to 0.36]	−0.56 [−1.4 to 0.33]	−0.29 [−1.2 to 0.88]	−0.15 [−0.88 to 0.99]	−0.78 [−1.7 to −0.13]	0.13 [−0.51 to 0.87]	−0.38 [−1.4 to 0.58]
Children breastfeeding, *n* (%)								
At 12 mo^3^	1328 (90.8)	125 (74.0)	194 (97.5)	191 (86.4)	196 (93.3)	202 (97.1)	191 (84.1)	229 (100)
At 18 mo^3^	1039 (71.0)	98 (58.0)	128 (64.3)	147 (66.5)	124 (59.0)	195 (93.8)	120 (52.9)	227 (99.1)
At 24 mo^3^	646 (44.6)	83 (49.4)	43 (22.4)	51 (23.2)	47 (22.7)	183 (88.0)	65 (28.6)	174 (76.3)
Children with underweight, *n* (%)								
At 12 mo^3^	202 (14.0)	1 (0.6)	15 (7.6)	21 (9.8)	26 (12.6)	48 (23.4)	73 (32.4)	18 (7.9)
At 18 mo^3^	230 (15.9)	3 (1.8)	16 (8.2)	18 (8.3)	33 (16.2)	62 (30.0)	76 (33.6)	22 (9.7)
At 24 mo^3^	257 (17.9)	3 (1.8)	14 (7.5)	16 (7.3)	46 (22.2)	67 (32.7)	82 (36.3)	29 (12.8)
Children with wasting, *n* (%)								
At 12 mo^3^	76 (5.3)	3 (1.8)	3 (1.5)	6 (2.8)	5 (2.4)	12 (6.3)	35 (15.6)	11 (4.8)
At 18 mo^3^	68 (4.7)	4 (2.4)	6 (3.1)	3 (1.4)	1 (0.5)	21 (10.1)	28 (12.4)	5 (2.2)
At 24 mo^3^	63 (4.4)	3 (1.8)	2 (1.1)	1 (0.5)	4 (2.0)	20 (9.8)	26 (11.5)	29 (12.8)
Children with stunting, *n* (%)								
At 12 mo^3^	384 (26.6)	5 (3.0)	58 (29.4)	56 (26.0)	102 (49.3)	68 (33.2)	71 (31.6)	24 (10.5)
At 18 mo^3^	545 (37.9)	5 (3.0)	82 (42.1)	73 (33.6)	60 (30.0)	96 (46.4)	104 (46.0)	45 (19.8)
At 24 mo^3^	551 (38.4)	6 (3.6)	72 (38.5)	76 (34.7)	60 (29.3)	100 (48.8)	100 (44.2)	52 (22.9)

1 Underweight was defined as when weight-for-age was < −2 *z* scores. Wasting was defined as when weight-for-length was < −2 *z* scores. Stunting was defined as when length-for-age was < −2 *z* scores. BGD, Dhaka, Bangladesh; BRF, Fortaleza, Brazil; INV, Vellore, India; MAL-ED, The Etiology, Risk Factors and Interactions of Enteric Infections and Malnutrition and the Consequences for Child Health and Development; NEB, Bhaktapur, Nepal; PEL, Loreto, Iquitos, Peru; SAV, Venda, South Africa; TZH, Haydom, Tanzania.

2 Kruskal–Wallis test was used to compare sites, *P* = 0.001.

3 Chi-square test, *P* < 0.0005.

At 24 mo of age, 61.9% of children with wasting were partially breastfed (chi-square test, *P* = 0.005). Prevalences of underweight and stunting were not significantly associated with breastfeeding status in any of the studied periods.

Energy and nutrient intakes from complementary foods were prospectively analyzed considering 3 time periods: 9–12 mo (**Table 2**), 13–18 mo (**Table 3**), and 19–24 mo (**Table 4**). Children with malnutrition presented significantly lower intakes of energy and zinc at 12, 18, and 24 mo of age, ranging from −16.4% to −25.9% for energy and −2.3% to −48.8% for zinc when compared with the intakes of children without underweight, wasting, and stunting. Other vitamin and mineral intakes were also consistently lower in the studied periods in children with underweight (niacin) and stunting (all of the vitamins and minerals, except for phosphorus at 24 mo). Fiber was higher in stunted children in the 3 time periods assessed.

**TABLE 2:** Intakes of energy and nutrients from complementary foods and nutritional status of children from the MAL-ED study at 12 mo of age^1^

	Underweight at 12 mo of age			Wasting at 12 mo of age			Stunting at 12 mo of age		
Nutrients	Yes (*n* = 199)	No (*n* = 1194)	Diff., %	Yes (*n* = 76)	No (*n* = 1316)	Diff., %	Yes (*n* = 373)	No (*n* = 1021)	Diff., %
Energy, kcal/d	376 [208–626]^2^	450 [256–730]	−16.4	376 [203–634]	443 [255–722]	−15.1	477 [268–713]	429 [237–722]	11.2
Macronutrients									
Protein, g/d	14.6 [12.5–16.2]^2^	15.2 [13.0–17.1]	−4.0	14.8 [13.1–16.0]	15.2 [12.8–17.0]	−2.6	14.6 [12.3–16.4]^2^	15.4 [13.1–17.2]	−5.2
Lipids, g/d	13.5 [10.7–15.4]	13.4 [10.3–16.0]	0.75	14.1 [12.4–16.5]^2^	13.3 [10.3–15.9]	6.0	12.1 [9.3–14.9]^2^	13.8 [11.0–16.2]	−12.3
Carbohydrates, g/d	79.5 [73.5–87.2]	78.2 [71.1–87.7]	1.7	77.2 [70.9–82.3]	78.4 [71.5–88.0]	−1.5	82.7 [74.5–91.2]^2^	77.2 [70.7–85.3]	7.1
Fiber, g/d	4.1 [3.2–5.5]	3.9 [2.3–6.0]	5.1	3.7 [2.9–4.9]	4.0 [2.3–6.0]	−7.5	4.7 [3.0–8.2]^2^	3.7 [2.3–5.3]	27.0
Vitamins									
Folate, μg/d	60.4 [42.5–71.4]	61.6 [42.3–83.9]	−2.0	61.9 [49.3–71.9]	61.2 [42.1–82.0]	1.1	54.5 [27.6–71.1]^2^	63.4 [47.2–85.7]	−14.0
Niacin, mg/d	2.7 [1.7–3.7]^2^	3.1 [2.0–4.0]	−12.9	2.5 [1.6–3.7]^2^	3.0 [2.0–4.0]	−16.7	2.7 [1.7–3.8]^2^	3.1 [2.1–4.1]	−12.9
Riboflavin, mg/d	0.44 [0.29–0.58]^2^	0.51 [0.35–0.63]	−13.7	0.43 [0.30–0.57]^2^	0.50 [0.34–0.63]	−14.0	0.46 [0.30–0.59]^2^	0.51 [0.35–0.63]	−9.8
Thiamin, mg/d	0.26 [0.17–0.32]^2^	0.28 [0.20–0.37]	−7.1	0.24 [0.15–0.32]	0.28 [0.20–0.36]	−14.3	0.26 [0.17–0.33]	0.28 [0.20–0.36]	−7.1
Vitamin A, μg/d	157 [83.3–245]^2^	208 [99.8–285]	−24.5	188 [101–254]	205 [95.4–279]	−8.3	155 [28.6–247]^2^	213 [117–288]	−27.2
Vitamin C, mg/d	14.0 [2.4–21.7]^2^	18.3 [5.1–33.1]	−23.5	15.7 [3.6–23.1]	17.5 [4.7–28.6]	−10.3	13.0 [3.7–22.7]^2^	19.2 [7.1–35.6]	−32.3
Minerals									
Calcium, mg/d	251 [159–329]^2^	278 [156–369]	−9.7	266 [189–336]	274 [152–364]	−2.9	226 [103–328]^2^	285 [175–373]	−20.7
Iron, mg/d	3.2 [1.8–4.2]^2^	3.8 [2.4–4.8]	−15.8	3.3 [2.1–4.1]	3.7 [2.4–4.7]	−10.8	3.4 [2.1–4.4]^2^	3.8 [4.7–2.4]	−10.5
Magnesium, mg/d	77.3 [65.8–87.9]	75.7 [59.4–90.4]	2.11	75.6 [62.8–83.1]	76.3 [60.5–90.9]	−0.9	82.2 [66.0–137]^2^	74.0 [58.7–86.3]	11.1
Potassium, mg/d	577 [470–664]	586 [470–706]	−1.5	586 [501–659]	585 [468–698]	0.17	564 [441–666]	589 [482–710]	−4.2
Phosphorus, mg/d	340 [279–435]^2^	384 [272–455]	−11.5	344 [289–435]	379 [273–452]	−9.2	348 [262–436]^2^	388 [280–458]	−10.3
Zinc, mg/d	2.4 [1.9–2.9]^2^	2.6 [2.0–3.1]	−7.7	2.4 [1.8–2.8]^2^	2.6 [2.0–3.0]	−7.7	2.4 [1.9–2.8]^2^	2.6 [2.0–3.1]	−7.7

1 Values are medians [IQRs], considering 24-h food recalls from 9–12 mo of age, unless otherwise indicated. Underweight was defined as when weight-for-age was < −2 *z* scores. Wasting was defined as when weight-for-length was < −2 *z* scores. Stunting was defined as when length-for-age was < −2 *z* scores. Diff. (%): differences in intake between the children with or without underweight, wasting, and stunting were expressed as percentages, considering children without underweight, wasting, and stunting as the reference value. MAL-ED, The Etiology, Risk Factors and Interactions of Enteric Infections and Malnutrition and the Consequences for Child Health and Development.

2 Mann–Whitney *U* test *P* < 0.05.

**TABLE 3:** Intakes of energy and nutrients from complementary foods and nutritional status of children from the MAL-ED study at 18 mo of age^1^

	Underweight at 18 mo of age			Wasting at 18 mo of age			Stunting at 18 mo of age		
Nutrients	Yes (*n* = 228)	No (*n* = 1210)	Diff., %	Yes (*n* = 68)	No (*n* = 1365)	Diff., %	Yes (*n* = 542)	No (*n* = 891)	Diff., %
Energy, kcal/d	548 [341–856]^2^	692 [419–959]	−20.8	508 [321–639]^2^	676 [412–954]	−24.9	672 [431–941]	662 [392–941]	1.5
Macronutrients									
Protein, g/d	20.0 [17.9–21.7]	20.3 [17.9–23.0]	−1.5	19.9 [18.0–21.9]	20.3 [17.9–22.8]	−2.0	19.5 [17.1–21.5]^2^	20.8 [18.4–23.8]	−6.3
Lipids, g/d	18.8 [16.5–21.2]	18.7 [14.6–21.8]	0.53	19.0 [17.7–21.3]	18.7 [14.7–21.8]	1.6	17.3 [13.6–20.4]^2^	19.2 [15.9–22.6]	−9.9
Carbohydrates, g/d	110 [104–117]	111 [101–121]	−0.90	110 [103–114]	111 [101–121]	−0.90	114 [106–125]^2^	109 [98.4–117]	4.6
Fiber, g/d	6.3 [4.9–7.8]^2^	5.8 [2.7–9.5]	8.6	5.8 [4.1–7.1]	5.9 [3.0–9.5]	−1.7	6.7 [4.5–11.0]^2^	5.5 [2.4–7.7]	21.8
Vitamins									
Folate, μg/d	84.4 [58.6–99.9]	88.2 [55.1–115]	−4.3	87.3 [74.1–98.2]	87.2 [54.3–112]	0.11	74.5 [40.5–98.3]^2^	93.6 [67.0–122]	−20.4
Niacin, mg/d	3.7 [2.8–5.2]^2^	4.4 [3.2–5.8]	−15.9	3.9 [2.9–5.3]	4.3 [3.1–5.7]	−9.3	3.8 [2.8–5.1]^2^	4.6 [3.3–6.2]	−17.4
Riboflavin, mg/d	0.68 [0.46–0.83]	0.68 [0.47–0.87]	0.00	0.69 [0.50–0.84]	0.68 [0.47–0.87]	1.5	0.62 [0.41–0.80]^2^	0.71 [0.50–0.90]	−12.7
Thiamin, mg/d	0.37 [0.26–0.48]^2^	0.42 [0.29–0.57]	−11.9	0.38 [0.26–0.49]	0.41 [0.28–0.53]	−7.3	0.37 [0.26–0.49]^2^	0.44 [0.30–0.60]	−15.9
Vitamin A, μg/d	222 [138–289]^2^	249 [138–342]	−10.8	247 [191–307]	245 [136–334]	0.82	198 [81.4–289]^2^	271 [182–378]	−26.9
Vitamin C, mg/d	18.7 [6.1–30.1]^2^	22.9 [7.5–42.4]	−18.3	21.4 [14.2–30.8]	22.4 [7.1–38.4]	−4.5	16.6 [0.37–29.9]^2^	25.1 [12.3–54.1]	−33.9
Minerals									
Calcium, mg/d	309 [225–414]	311 [166–433]	−0.64	310 [255–420]	310 [173–426]	0.00	275 [139–377]^2^	336 [200–503]	−18.2
Iron, mg/d	4.6 [3.2–5.8]^2^	5.3 [3.6–6.6]	−13.2	4.4 [3.1–6.0]	5.2 [3.6–6.5]	−15.4	4.8 [3.2–6.2]^2^	5.3 [3.8–6.9]	−9.4
Magnesium, mg/d	108 [92.7–124]	105 [80.6–124]	2.9	107 [90.4–115]	104 [83.3–124]	2.9	112 [90.7–192]^2^	101 [79.1–118]	10.9
Potassium, mg/d	801 [701–904]	790 [642–929]	1.4	813 [730–905]	790 [648–926]	2.9	764 [632–879]^2^	812 [666–973]	−5.9
Phosphorus, mg/d	465 [391–541]	468 [358–562]	−0.64	467 [390–542]	466 [363–559]	0.21	451 [355–535]^2^	483 [371–586]	−6.6
Zinc, mg/d	3.3 [2.8–3.7]^2^	3.5 [2.9–4.2]	−5.7	3.2 [2.8–3.7]^2^	3.5 [2.8–4.1]	−8.6	3.3 [2.7–3.8]^2^	3.6 [2.9–4.5]	−8.3

1 Values are medians [IQRs], considering 24-h food recalls from 13 to 18 mo of age, unless otherwise indicated. Underweight was defined as when weight-for-age was < −2 *z* scores. Wasting was defined as when weight-for-length was < −2 *z* scores. Stunting was defined as when length-for-age was < −2 *z* scores. Diff. (%): differences in intake between the children with or without underweight, wasting, and stunting were expressed as percentages, considering children without underweight, wasting, and stunting as the reference value. MAL-ED, The Etiology, Risk Factors and Interactions of Enteric Infections and Malnutrition and the Consequences for Child Health and Development.

2 Mann–Whitney *U* test *P* < 0.05.

**TABLE 4:** Intakes of energy and nutrients from complementary foods and nutritional status of children from the MAL-ED study at 24 mo of age^1^

	Underweight at 24 mo of age			Wasting at 24 mo of age			Stunting at 24 mo of age		
Nutrients	Yes (*n* = 257)	No (*n* = 1179)	Diff., %	Yes (*n* = 63)	No (*n* = 1371)	Diff., %	Yes (*n* = 551)	No (*n* = 883)	Diff., %
Energy, kcal/d	771 [482–1050]^2^	959 [673–1184]	−19.6	695 [395–994]^2^	938 [640–1170]	−25.9	925 [621–1171]	929 [617–1161]	−0.43
Macronutrients									
Protein, g/d	26.0 [23.6–28.1]^2^	27.0 [23.4–30.8]	−3.7	25.7 [23.8–27.9]	26.5 [23.4–30.3]	−3.0	25.4 [22.3–28.5]^2^	27.2 [24.2–31.9]	−6.6
Lipids, g/d	13.5 [10.6–15.4]	13.4 [10.4–16.1]	0.75	25.1 [23.0–28.3]	24.0 [19.0–28.1]	4.6	22.0 [14.7–26.3]^2^	25.1 [20.8–29.1]	−12.4
Carbohydrates, g/d	146 [138–157]	145 [131–157]	0.69	145 [136–149]	145 [133–157]	0.00	150 [141–169]^2^	142 [129–153]	5.6
Fiber, g/d	8.4 [6.4–11.3]^2^	7.7 [3.7–13.1]	9.1	7.2 [6.0–9.2]	7.9 [4.2–13.1]	−8.9	9.5 [6.4–15.7]^2^	6.9 [3.3–10.6]	37.7
Vitamins									
Folate, μg/d	101 [71.7–118]^2^	109 [69.2–150]	−7.3	105 [86.9–117]	107 [69.0–142]	−1.9	96.3 [52.7–120]^2^	115 [85.6–158]	−16.3
Niacin, mg/d	5.0 [3.8–6.3]^2^	5.5 [4.2–8.0]	−9.1	5.0 [3.5–6.3]	5.4 [4.1–7.5]	−7.4	5.2 [4.0–6.6]^2^	5.6 [4.1–8.3]	−7.1
Riboflavin, mg/d	0.74 [0.48–0.90]	0.78 [0.55–1.1]	−5.1	0.77 [0.51–0.91]	0.77 [0.54–1.0]	0.00	0.72 [0.45–0.90]^2^	0.81 [0.59–1.1]	−11.1
Thiamin, mg/d	0.50 [0.35–0.60]	0.52 [0.37–0.76]	−3.9	0.49 [0.34–0.58]	0.52 [0.37–0.71]	−5.8	0.50 [0.35–0.62]^2^	0.54 [0.37–0.77]	−7.4
Vitamin A, μg/d	258 [151–325]	271 [165–373]	−4.8	286 [221–349]	268 [163–361]	6.7	225 [106–314]^2^	294 [202–419]	−23.5
Vitamin C, mg/d	23.6 [6.6–35.0]	25.7 [9.2–53.7]	−8.2	29.7 [8.9–37.2]	25.2 [8.9–47.4]	17.9	18.4 [2.5–35.1]^2^	29.5 [13.2–61.7]	−37.6
Minerals									
Calcium, mg/d	331 [216–416]	312 [162–491]	6.1	346 [290–416]^2^	313 [165–481]	10.5	267 [113–376]^2^	357 [204–567]	−25.2
Iron, mg/d	5.7 [3.7–7.2]^2^	6.3 [4.3–8.9]	−9.5	5.5 [2.9–6.9]	6.2 [4.3–8.6]	−11.3	6.2 [4.4–8.2]	6.3 [4.2–9.2]	−1.6
Magnesium, mg/d	137 [124–162]^2^	130 [105–156]	5.4	131 [115–152]	132 [107–158]	−0.8	141 [120–265]^2^	127 [102–148]	11.0
Potassium, mg/d	987 [877–1097]	966 [826–1131]	2.2	987 [867–1076]	969 [835–1127]	1.9	931 [809–1063]^2^	1000 [860–1201]	−6.9
Phosphorus, mg/d	564 [490–623]	546 [422–648]	3.3	564 [504–622]	548 [431–645]	2.9	541 [433–615]	552 [432–673]	−2.0
Zinc, mg/d	4.1 [3.6–4.5]^2^	4.3 [3.5–5.6]	−4.6	4.1 [3.4–4.5]	4.3 [3.5–5.2]	−4.7	4.2 [3.4–4.6]^2^	4.3 [3.5–6.2]	−2.3

1 Values are medians [IQRs], considering 24-h food recalls from 19 to 24 mo of age, unless otherwise indicated. Underweight was defined as when weight-for-age was < −2 *z* scores. Wasting was defined as when weight-for-length was < −2 *z* scores. Stunting was defined as when length-for-age was < −2 *z* scores. Diff. (%): differences in intake between the children with or without underweight, wasting, and stunting were expressed as percentages, considering children without underweight, wasting, and stunting as the reference value. MAL-ED, The Etiology, Risk Factors and Interactions of Enteric Infections and Malnutrition and the Consequences for Child Health and Development.

2 Mann–Whitney *U* test *P* < 0.05.

The logistic regressions showed higher energy intakes were associated with decreased risk of underweight at 12 (AOR: 0.90; 95% CI: 0.84, 0.96) and 24 mo (AOR: 0.91; 95% CI: 0.86, 0.96), and wasting at 18 (AOR: 0.91; 95% CI: 0.83, 0.99) and 24 mo (AOR: 0.83; 95% CI: 0.74, 0.92). Higher zinc intakes were associated with decreased risk of underweight (AOR: 0.12; 95% CI: 0.03, 0.55) and wasting (AOR: 0.10; 95% CI: 0.04, 0.92) at 12 mo, and wasting (AOR: 0.05; 95% CI: 0.00, 0.76) at 24 mo (**Table 5**).

**TABLE 5:** Logistic regression models of nutrient intakes from complementary foods and the risk of undernutrition at 12, 18, and 24 mo of age in children from the MAL-ED cohort^1^

	Underweight		Wasting		Stunting	
Variables	OR (95% CI)	AOR (95% CI)	OR (95% CI)	AOR (95% CI)	OR (95% CI)	AOR (95% CI)
12 mo						
Energy	0.96 (0.94, 0.98)	0.90 (0.84, 0.96)	0.97 (0.94, 1.00)	0.92 (0.85, 1.01)	0.99 (0.98, 1.01)	1.01 (0.97, 1.05)
Protein intake	0.70 (0.55, 0.90)	0.55 (0.28, 1.10)	0.79 (0.54, 1.15)	0.78 (0.31, 1.20)	0.61 (0.54, 0.73)	0.76 (0.49, 1.19)
Vitamin A intake	0.93 (0.90, 0.95)	1.05 (0.99, 1.12)	0.94 (0.90, 0.99)	1.01 (0.91, 1.12)	0.93 (0.91, 0.95)	1.16 (0.81, 1.65)
Iron intake	0.52 (0.39, 0.68)	0.79 (0.45, 1.37)	0.65 (0.43, 0.96)	1.15 (0.54, 2.45)	0.59 (0.49, 0.71)	1.02 (0.98, 1.06)
Zinc intake	0.38 (0.25, 0.59)	0.12 (0.03, 0.55)	0.46 (0.24, 0.88)	0.19 (0.04, 0.92)	0.39 (0.29, 0.54)	0.81 (0.35, 1.91)
18 mo						
Energy	0.95 (0.93, 0.97)	0.95 (0.90, 1.00)	0.92 (0.89, 0.96)	0.91 (0.83, 0.99)	1.00 (0.99, 1.02)	0.99 (0.95, 1.02)
Protein intake	0.77 (0.61, 0.98)	1.40 (0.75, 2.64)	0.86 (0.56, 1.30)	1.00 (0.33, 3.03)	0.46 (0.37, 0.56)	0.78 (0.54, 1.13)
Vitamin A intake	0.95 (0.93, 0.97)	1.00 (0.95, 1.06)	0.98 (0.94, 1.02)	1.01 (0.92, 1.11)	0.93 (0.91, 0.95)	1.04 (1.00, 1.07)
Iron intake	0.58 (0.46, 0.73)	1.49 (0.92, 2.39)	0.62 (0.42, 0.92)	1.08 (0.42, 2.75)	0.55 (0.46, 0.66)	0.95 (0.68, 1.32)
Zinc intake	0.49 (0.35, 0.70)	0.43 (0.11, 1.79)	0.59 (0.33, 1.07)	0.32 (0.04, 2.43)	0.38 (0.29, 0.50)	1.06 (0.49, 2.28)
24 mo						
Energy	0.94 (0.92, 0.96)	0.91 (0.86, 0.96)	0.91 (0.88, 0.95)	0.83 (0.74, 0.92)	1.00 (0.98, 1.02)	0.98 (0.95, 1.02)
Protein intake	0.62 (0.49, 0.79)	1.12 (0.61, 2.05)	0.69 (0.45, 1.06)	0.95 (0.24, 3.72)	0.46 (0.38, 0.56)	1.03 (0.71, 1.50)
Vitamin A intake	0.96 (0.93, 0.98)	1.03 (0.99, 1.08)	0.98 (0.94, 1.02)	1.10 (1.00, 1.21)	0.93 (0.91, 0.94)	1.01 (0.98, 1.04)
Iron intake	0.64 (0.53, 0.78)	1.21 (0.75, 1.96)	0.59 (0.42, 0.84)	0.66 (0.22, 1.94)	0.76 (0.66, 0.88)	1.37 (0.98, 1.90)
Zinc intake	0.52 (0.38, 0.71)	1.12 (0.74, 1.68)	0.49 (0.28, 0.86)	0.05 (0.00, 0.76)	0.47 (0.36, 0.59)	0.53 (0.23, 1.24)

1 Crude ORs were calculated by logistic regressions in bivariate analyses, exploring the effect of a single nutrient intake on the studied outcomes. Nutrients showing a correlation (Pearson's *r* > 0.7) were excluded from the adjusted models. Adjustment variables in all models were sex; country of the site; length-for-age at enrollment (within 17 d of birth); breastfeeding at 12, 18, or 24 mo (yes/no); and bacterial, viral, and parasite burdens. Energy and nutrient intakes were calculated considering 24-h recalls from 9–12 mo for the 12-mo models, 13–18 mo for the 18-mo models, and 19–24 mo for the 24-mo models. Adjusted nutrient intakes were approximated to normal distributions before entering the models using their square root. Underweight was defined as when weight-for-age was < −2 *z* scores. Wasting was defined as when weight-for-length was < −2 *z* scores. Stunting was defined as when length-for-age was < −2 *z* scores. MAL-ED, The Etiology, Risk Factors and Interactions of Enteric Infections and Malnutrition and the Consequences for Child Health and Development.

Because energy and zinc intakes were associated with lower risk of underweight and wasting, we evaluated these intakes further within each site, considering the 3 time periods: 9–12 mo, 13–18 mo, and 19–24 mo (**Supplemental Figures 1–4**). Children from the South Asian sites (BGD, INV, and NEB) presented lower energy and zinc intakes than those from Latin American and African sites (Supplemental Figures 1–4). BRF, PEL, SAV, and TZH presented similar energy intakes (Supplemental Figures 1, 3), and the Brazilian (BRF) and Peruvian (PEL) sites presented higher zinc intakes, when compared with the other sites (Supplemental Figures 2, 4).

## Discussion

Most of the undernutrition in low- and middle‐income countries happens during the 1000-d period that encompasses pregnancy and the child's first 2 y after birth (29), and evidence shows that a substantial proportion of undernutrition occurs during the complementary feeding period (6–23 mo) (30). This study has shown the association of specific nutrient intakes from complementary feeding and the development of undernutrition in terms of its 3 primary anthropometric outcomes from a prospective analysis. Key findings demonstrate that greater total energy and zinc intakes from non-breast-milk foods are associated with protection against undernutrition.

For the first time to our knowledge, quantitative longitudinal data are shown comparing usual nutrient intakes from complementary feeding between children with and without underweight, wasting, and stunting from 9 to 24 mo. As expected, these intakes were considerably lower in children presenting underweight, wasting, and stunting. Studies characterizing energy and nutrient intake through 24-h food recalls in children presenting undernutrition have been done mostly in sectional designs or using few repetitions of the 24-h recall, within a 3-mo age period, which compromises usual intake determination. In the present study, we were able to demonstrate lower intakes of energy, macronutrients, 6 vitamins, and 6 minerals in children with undernutrition. Interestingly, fiber intakes were significantly higher in children presenting stunting.

Worldwide representative human data of quantitative nutrient intake from complementary feeding in children at 6–24 mo of age are lacking. Studies have characterized complementary feeding nationally using the WHO's core indicators, which make data collection easier. These data are available within the UNICEF global database, and have been recently revised (31). Other studies have addressed quantitative nutrient intake from complementary feeding in local communities, but none were able to associate these intakes as nutritional status determinants, in a cross-country analysis (9–12). These dietary intake analyses were also limited in preclinical studies to induce undernutrition. In these studies, most commonly consumed foods in undernourished children were assessed and used to derive animal diets, but quantitative assessments of children's nutrient intakes were not performed (32, 33).

In the present study, we were able to associate energy and nutrient intakes from complementary feeding to nutritional outcomes. Our findings show that in the sites where undernutrition was more prevalent—in the Eastern African site (TZH) and South Asian sites (BGV, INV, and NEB)—zinc intakes from complementary feeding were lower and in the South Asian sites (BGV, INV, and NEB) these intakes tended to not increase over time. Also, in the South Asian sites, where energy intakes from complementary feeding were lower from 9 to 24 mo of age than at the other studied sites, undernutrition was more prevalent.

In South Asia, <3 in 5 infants aged 6–8 mo consume soft, semisolid, or solid foods, indicating late initiation of complementary feeding. In this region, complementary foods for children aged 6–23 mo are primarily cereal‐based, lacking the essential growth‐promoting nutrients provided by fruits, vegetables, and foods of animal origin (34). These characteristics of low-density meals, lacking animal sources of foods, might explain the lower energy and zinc intakes from complementary feeding found in the South Asian sites (BGD, INV, and NEB) in the present study. These results reinforce that poor complementary feeding may play a crucial role in the development of undernutrition, and studies should address how to improve access to safe and healthy foods for children from the studied sites.

Overall, children from the Latin American sites in the study (BRF and PEL) and those from Eastern and South Africa (TZH and SAV) presented similar energy intakes from complementary feeding. White et al. (31) found that the 2 regions with the best indicators of complementary feeding were East Asia/the Pacific and Latin America/the Caribbean, the same 2 regions with the most extensive improvements in stunting between 1990 and 2015, at 75% and 55%, respectively (35).

Children from BRF and SAV presented higher zinc intakes from complementary feeding than the other studied sites. In Brazil, as previously reported (36), these results could be attributable to increased consumption of industrialized infant foods in the BRF site, especially cereals fortified with zinc, iron, and vitamins. In SAV, the commonly consumed maize meal is fortified with zinc and other micronutrients, and industrialized fortified infant cereals are also available (37). These commercially fortified foods were commonly seen in the 24-h recalls from children in BRF and SAV, but were not frequently reported as consumed in the other studied sites.

Better complementary feeding practices predict better linear growth outcomes, and our study reinforces that energy and zinc from complementary food are lower in children with stunting. For stunted children, we also found higher fiber intakes in the 3 time points evaluated. Zinc supplementation studies have shown this nutrient has a particularly positive effect on growth (38), and marginal zinc deficiency and suboptimal zinc status have been associated with stunting (39). Although the cause may be inadequate dietary zinc intake (39), which was present in our stunted children, inhibitors of zinc absorption are a common causative factor. Phytate, present in staple foods like cereals, corn, and rice, has a strong negative effect on zinc absorption (40). Thus, the higher fiber intakes found in the stunted children from the present study might be an additional concern relating to zinc bioavailability in composite meals of these children.

Nutrient intake recommendations may vary according to diet characteristics that determine bioavailability (41). In the present study, dietary intakes were not compared to recommendations, rather we assessed intakes from complementary foods. Diet bioavailability varied among sites, and the probability of adequacy, considering breast-milk intake, was assessed in a previous analysis aiming to characterize the adequacy of dietary intake of study children (20). In the present study, we hypothesized that lower energy and nutrient intakes coming from complementary feeding in children from 9 to 24 mo of age would increase the risk of undernutrition in this life period. This hypothesis was corroborated by comparing usual intakes from complementary food in children with and without undernutrition. Further studies should assess how complementary feeding bioavailability could affect even more the development of undernutrition.

One of the limitations of our results is that the data are not nationally representative in each country. Nevertheless, the findings from the present study may apply to other communities within the studied countries. The potential for type I errors could be a limitation considering the multiple nutrient exposures assessed across outcomes. We began to collect dietary recalls at 9 mo of age, although introduction of non-breast-milk foods began well before 6 mo for most study infants. Strengths of our study are the prospective data collection with 24-h dietary recalls, which allowed for the collection of 15–17 dietary intake recalls per child, with equivalent data collection procedures across the sites, and the use of local food composition tables. This is the first study, to the best of our knowledge, to use this kind of approach to understand complementary feeding and its associations with nutritional outcomes.

Further studies should also explore pathways from direct host metabolism to effects on the microbiome or even possible pathogen virulence expression as they relate to energy and zinc intake associations with underweight, wasting, and stunting. Analyzing complementary feeding intake from a usual quantitative nutrient intake perspective is an advantage of the MAL-ED cohort study protocol. In addition, assessing nutrient intake at 3 different time points has allowed the observation of lower energy and zinc intakes in children with undernutrition during the first 2 y of life.

In summary, these results show the importance of nutrient intake from complementary feeding for the prevention of undernutrition in terms of its 3 primary anthropometric outcomes. Higher energy and zinc intakes in complementary feeding were associated with decreased risk of undernutrition. Data suggest these are complementary feeding characteristics to be improved across sites. More research should be conducted to support governments to identify national constraints, and to design and to implement specific programs to improve complementary feeding.

## Supplementary Material

nxaa271_Supplemental_File

## Notes

MAL-ED (The Etiology, Risk Factors and Interactions of Enteric Infections and Malnutrition and the Consequences for Child Health and Development Project) was a collaborative project supported by the Bill & Melinda Gates Foundation, the Foundation for the NIH, and NIH/Fogarty International Center grant OPP47075.

Author disclosures: The authors report no conflicts of interest.

Supplemental Figures 1–4 are available from the “Supplementary data” link in the online posting of the article and from the same link in the online table of contents at https://academic.oup.com/jn/.

Abbreviations used: BGD, Dhaka, Bangladesh; BRF, Fortaleza, Brazil; INV, Vellore, India; JHSPH, Johns Hopkins University Bloomberg School of Public Health; MAL-ED, The Etiology, Risk Factors and Interactions of Enteric Infections and Malnutrition and the Consequences for Child Health and Development; NEB, Bhaktapur, Nepal; PEL, Loreto, Iquitos, Peru; SAV, Venda, South Africa; TZH, Haydom, Tanzania.
