# Supplementary material for: Higher Energy and Zinc Intakes from Complementary Feeding Are Associated with Decreased Risk of Undernutrition in Children from South America, Africa, and Asia
Source: J Nutr. 2020 Sep 16;151(1):170–8. doi: 10.1093/jn/nxaa271 (PMC7779220; doi:10.1093/jn/nxaa271)
Supplement: nxaa271_Supplemental_File [file nxaa271_supplemental_file.pdf]

Higher energy and zinc intakes from complementary feeding are associated with decreased risk for undernutrition in children from South America, Africa and Asia

Maciel et al

Online Supplementary Material

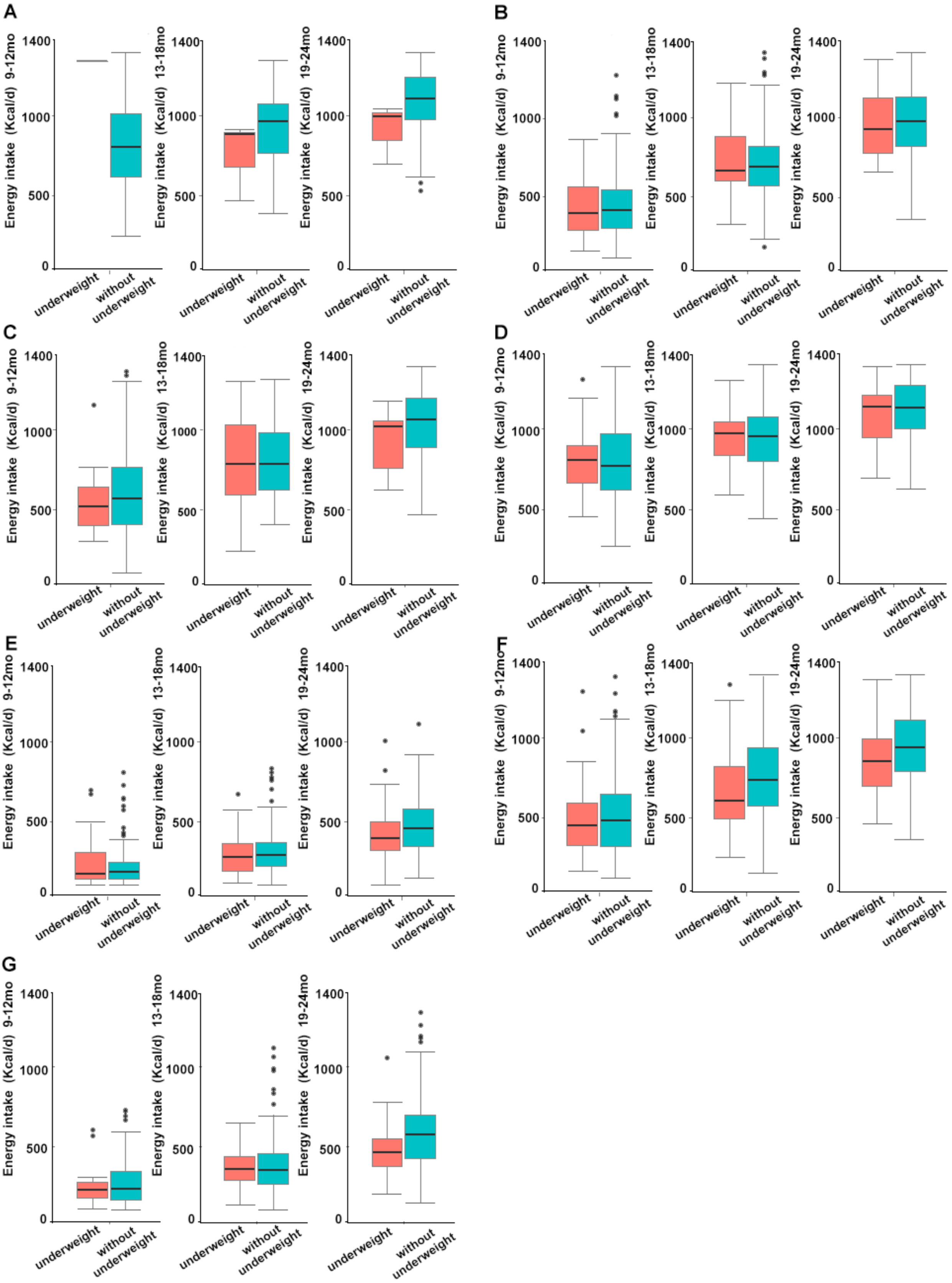

Supplemental Figure 1. Energy intake (Kcal/d) considering children with and without underweight at 12, 18 and 24 months in the MAL-ED sites in **A)** Fortaleza, Brazil (at 12

months: n = 1 child with underweight, n = 168 without underweight; at 18 months: n = 4 children with underweight, n = 163 without underweight; at 24 months: n = 3 with underweight, n = 162 without underweight); **B)** Loreto, Peru (at 12 months: n = 15 children with underweight, n = 182 without underweight; at 18 months: n = 6 children with underweight, n = 189 without underweight; at 24 months: n = 14 with underweight, n = 173 without underweight) ; **C)** Venda, South Africa (at 12 months: n = 21 children with underweight, n = 194 without underweight; at 18 months: n = 3 with underweight, n = 214 without underweight; at 24 months: n = 16 child with underweight, n = 203 without underweight); **D)** Haydon, Tanzania (at 12 months: n = 26 children with underweight, n = 180 without underweight; at 18 months: n = 1 child with underweight, n = 199 without underweight; at 24 months: n = 46 with underweight, n = 161 without underweight); **E)** Dhaka, Bangladesh (at 12 months: n = 48 children with underweight, n = 157 without underweight; at 18 months: n = 21 with underweight, n = 186 without underweight; at 24 months: n = 67 with underweight, n = 138 without underweight); **F)** Vellore, India (at 12 months: n = 73 with underweight, n = 152 without underweight; at 18 months: n = 28 with underweight, n = 198 without underweight; at 24 months: n = 82 with underweight, n = 144 without underweight); **G)** Bhaktapur, Nepal (at 12 months: n = 18 children with underweight, n = 210 without underweight; at 18 months: n = 5 with underweight, n = 222 without underweight; at 24 months: n = 29 with underweight, n = 198 without underweight). Total n = 1463 children, with dietary data collected using monthly 24h recalls. Underweight was defined when weight-for-age was < -2 z scores. Intakes were corrected for within- and between-person variance with the ANOVA test. Median (Q1, Q3) values are shown for each period, and the Mann-Whitney’s test was used to compare children with and without underweight in the time points.

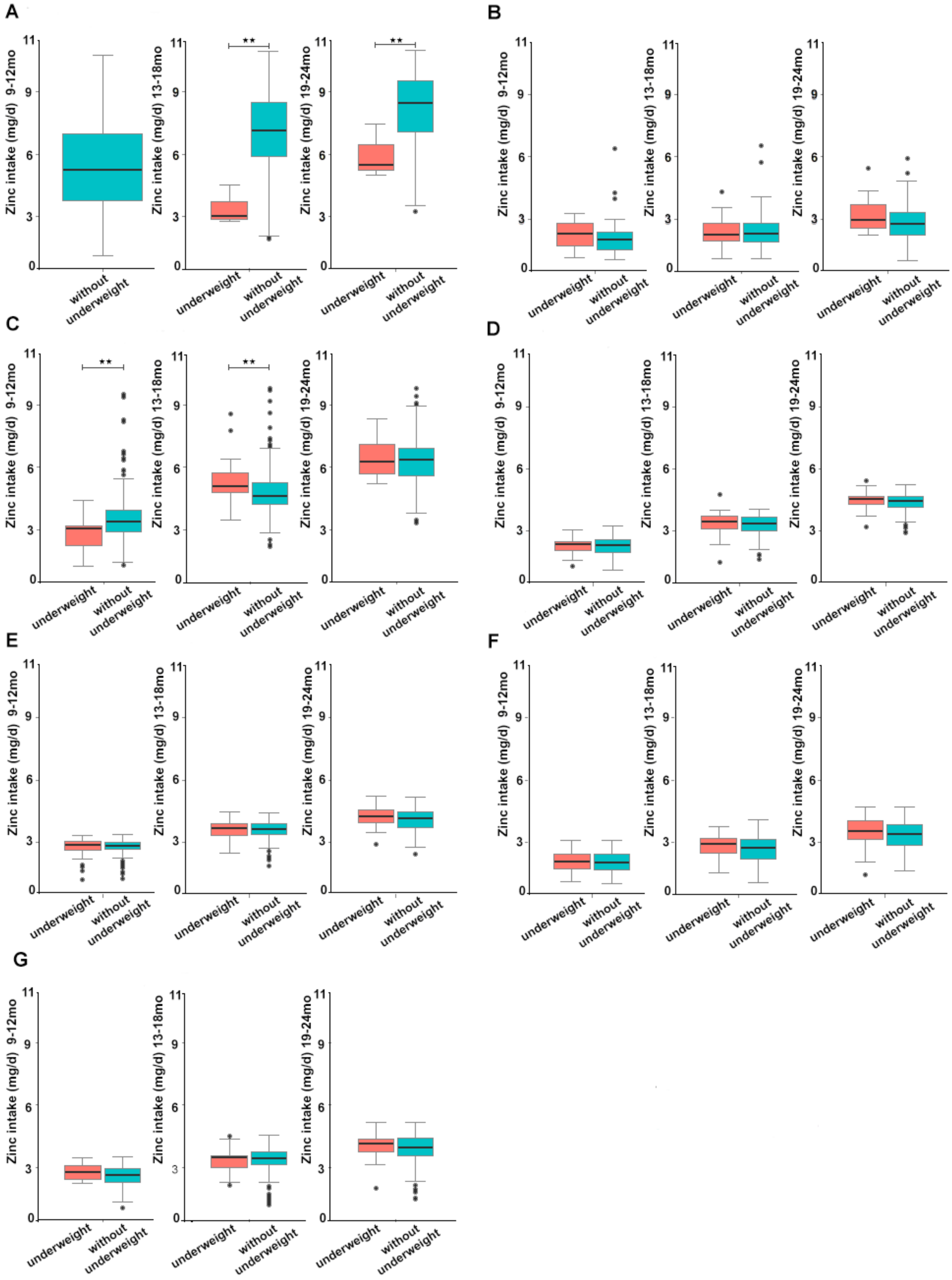

Supplemental Figure 2. Zinc intake (mg/d) considering children with and without underweight at 12, 18 and 24 months in the MAL-ED sites in **A)** Fortaleza, Brazil (at 12 months: n = 1 child with underweight, n = 168 without underweight; at 18 months: n = 4

children with underweight, n = 163 without underweight; at 24 months: n = 3 with underweight, n = 162 without underweight); **B)** Loreto, Peru (at 12 months: n = 15 children with underweight, n = 182 without underweight; at 18 months: n = 6 children with underweight, n = 189 without underweight; at 24 months: n = 14 with underweight, n = 173 without underweight) ; **C)** Venda, South Africa (at 12 months: n = 21 children with underweight, n = 194 without underweight; at 18 months: n = 3 with underweight, n = 214 without underweight; at 24 months: n = 16 child with underweight, n = 203 without underweight); **D)** Haydon, Tanzania (at 12 months: n = 26 children with underweight, n = 180 without underweight; at 18 months: n = 1 child with underweight, n = 199 without underweight; at 24 months: n = 46 with underweight, n = 161 without underweight); **E)** Dhaka, Bangladesh (at 12 months: n = 48 children with underweight, n = 157 without underweight; at 18 months: n = 21 with underweight, n = 186 without underweight; at 24 months: n = 67 with underweight, n = 138 without underweight); **F)** Vellore, India (at 12 months: n = 73 with underweight, n = 152 without underweight; at 18 months: n = 28 with underweight, n = 198 without underweight; at 24 months: n = 82 with underweight, n = 144 without underweight); **G)** Bhaktapur, Nepal (at 12 months: n = 18 children with underweight, n = 210 without underweight; at 18 months: n = 5 with underweight, n = 222 without underweight; at 24 months: n = 29 with underweight, n = 198 without underweight). Total n = 1463 children, with dietary data collected using monthly 24h recalls. Underweight was defined when weight-for-age was < -2 z scores. Intakes were corrected for within- and between-person variance with the ANOVA test. Adjustment for the total reported energy intake was performed for zinc intake using the residual method. Median (Q1, Q3) values are shown for each period, and the Mann-Whitney's test was used to compare children with and without underweight in the time points. **$p < 0.01$.

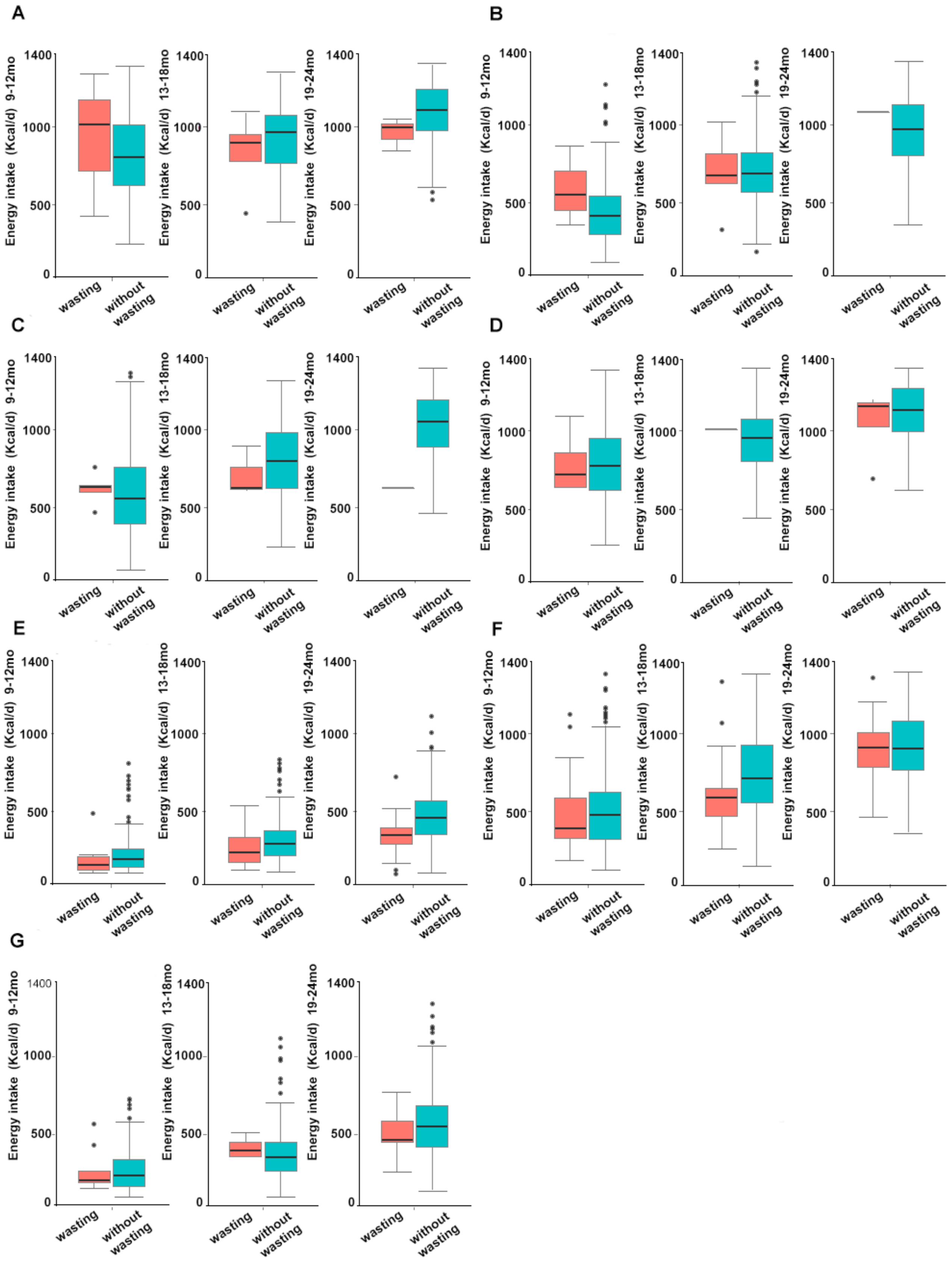

Supplemental Figure 3. Energy intake (Kcal/d) considering children with and without wasting at 12, 18 and 24 months in the MAL-ED sites in **A)** Fortaleza, Brazil (at 12 months: n = 3

children with wasting, n = 166 without wasting; at 18 months: n = 4 children with wasting, n = 163 without wasting; at 24 months: n = 3 children with wasting, n = 162 without wasting); **B)** Loreto, Peru (at 12 months: n = 3 children with wasting, n = 194 without wasting; at 18 months: n = 6 children with wasting, n = 189 without wasting; at 24 months: n = 2 children with wasting, n = 185 without wasting); **C)** Venda, South Africa (at 12 months: n = 6 children with wasting, n = 209 without wasting; at 18 months: n = 3 children with wasting, n 214 without wasting; at 24 months: n = 1 child with wasting, n = 218 without wasting); **D)** Haydon, Tanzania (at 12 months: n = 5 children with wasting, n = 200 without wasting; at 18 months: n = 1 with wasting, n= 199 without wasting; at 24 months: n = 4 children with wasting, n = 201 without wasting); **E)** Dhaka, Bangladesh (at 12 months: n = 12 children with wasting, n = 192 without wasting; at 18 months: n = 21 with wasting, n = 186 without wasting; at 24 months: 20 children with wasting, n = 185 without wasting); **F)** Vellore, India (at 12 months: n = 35 children with wasting, n = 190 without wasting; at 18 months: n = 28 with wasting, n = 198 without wasting; at 24 months: n = 26 children with wasting, n = 200 without wasting); **G)** Bhaktapur, Nepal (at 12 months: n = 11 children with wasting, n = 217 without wasting; at 18 months: n = 5 children with wasting, n = 222 without wasting; at 24 months: n = 29 children with wasting, n = 198 without wasting). Total n = 1463 children, with dietary data collected using monthly 24h recalls. Wasting was defined when weight-for-length was < -2 z scores. Intakes were corrected for within- and between-person variance with the ANOVA test. Median (Q1, Q3) values are shown for each period, and the Mann-Whitney's test was used to compare children with and without wasting in the time points.

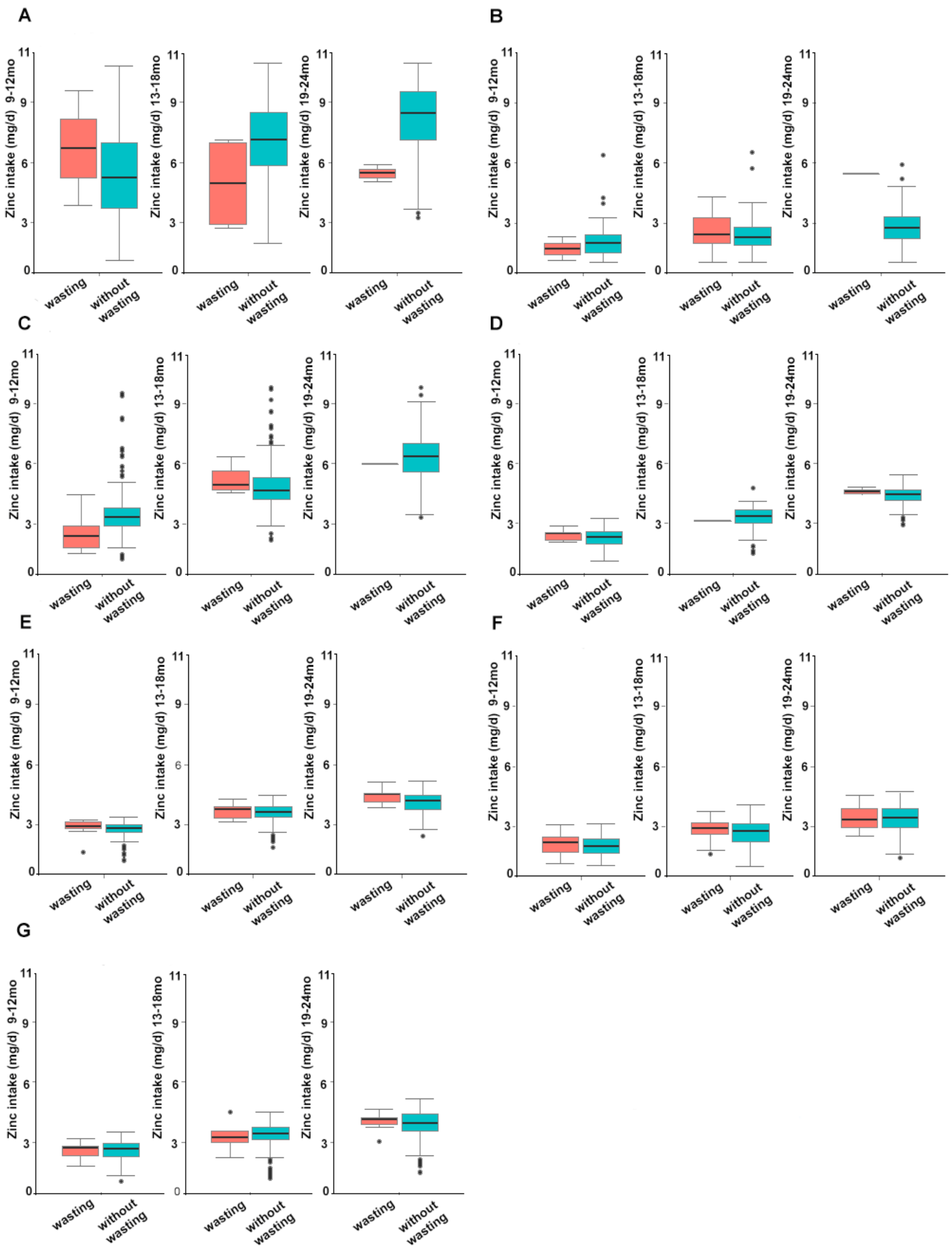

Supplemental Figure 4. Zinc intake (mg/d) considering children with and without wasting at 12, 18 and 24 months in the MAL-ED sites in **A)** Fortaleza, Brazil (at 12 months: n = 3 children with wasting, n = 166 without wasting; at 18 months: n = 4 children with wasting, n

= 163 without wasting; at 24 months: n = 3 children with wasting, n = 162 without wasting); **B)** Loreto, Peru (at 12 months: n = 3 children with wasting, n = 194 without wasting; at 18 months: n = 6 children with wasting, n = 189 without wasting; at 24 months: n = 2 children with wasting, n = 185 without wasting); **C)** Venda, South Africa (at 12 months: n = 6 children with wasting, n = 209 without wasting; at 18 months: n = 3 children with wasting, n 214 without wasting; at 24 months: n = 1 child with wasting, n = 218 without wasting); **D)** Haydon, Tanzania (at 12 months: n = 5 children with wasting, n = 200 without wasting; at 18 months: n = 1 with wasting, n= 199 without wasting; at 24 months: n = 4 children with wasting, n = 201 without wasting); **E)** Dhaka, Bangladesh (at 12 months: n = 12 children with wasting, n = 192 without wasting; at 18 months: n = 21 with wasting, n = 186 without wasting; at 24 months: 20 children with wasting, n = 185 without wasting); **F)** Vellore, India (at 12 months: n = 35 children with wasting, n = 190 without wasting; at 18 months: n = 28 with wasting, n = 198 without wasting; at 24 months: n = 26 children with wasting, n = 200 without wasting); **G)** Bhaktapur, Nepal (at 12 months: n = 11 children with wasting, n = 217 without wasting; at 18 months: n = 5 children with wasting, n = 222 without wasting; at 24 months: n = 29 children with wasting, n = 198 without wasting). Total n = 1463 children, with dietary data collected using monthly 24h recalls. Wasting was defined when weight-for-length was < -2 z scores. Intakes were corrected for within- and between-person variance with the ANOVA test. Adjustment for the total reported energy intake was performed for zinc intake using the residual method. Median (Q1, Q3) values are shown for each period, and the Mann-Whitney's test was used to compare children with and without wasting in the time points.
